# Structure equation model and neural network analyses to predict coronary artery lesions in Kawasaki disease: a single-centre retrospective study

**DOI:** 10.1038/s41598-020-68657-0

**Published:** 2020-07-17

**Authors:** Junji Azuma, Takehisa Yamamoto, Motoaki Nitta, Yasuhiro Hasegawa, Eri Kijima, Tsunesuke Shimotsuji, Yoshimi Mizoguchi

**Affiliations:** Department of Paediatrics, Minoh City Hospital, 5-7-1 Kayano, Minoh, Osaka 562-0014 Japan

**Keywords:** Predictive markers, Paediatric research, Vasculitis syndromes, Statistics

## Abstract

A new method to predict coronary artery lesions (CALs) in Kawasaki disease (KD) was developed using a mean structure equation model (SEM) and neural networks (Nnet). There were 314 admitted children with KD who met at least four of the six diagnostic criteria for KD. We defined CALs as the presence of a maximum z score of ≥ 3.0. The SEM using age, sex, intravenous immunoglobulin resistance, number of steroid pulse therapy sessions, C-reactive protein level, and urinary β2-microglobulin (u-β2MG/Cr) values revealed a perfect fit based on the root mean square error of approximation with an *R*^2^ value of 1.000 and the excellent discrimination of CALs with a sample score (SS) of 2.0 for a latent variable. The Nnet analysis enabled us to predict CALs with a sensitivity, specificity and c-index of 73%, 99% and 0.86, respectively. This good and simple statistical model that uses common parameters in clinical medicine is useful in deciding the appropriate therapy to prevent CALs in Japanese KD patients.

## Introduction

Kawasaki disease (KD) was reported for the first time by Tomisaku Kawasaki in 1967^1^. KD is a type of vasculitis in medium-sized arteries such as the coronary artery^2^, leading to coronary artery lesions (CALs) as major complications. The cause of KD is still unknown and several pathogenic etiologies for KD have been proposed including the Epstein Barr (EB) virus^3^, the super antigen for staphylococci^4^, lipopolysaccharide bound to CD14 on neutrophils^5^, environmental factors^6^ and abnormal innate immunity^7^. Also, several genes associated with KD have been reported^8,9^. However, their roles in the pathogenesis of CALs are still unclear. The recent advance in this field was the finding of responsive proteins associated with the occurrence of KD^10^. However, to date, the roles of these proteins in the occurrence of CALs are unclear.

The classic intravenous immunoglobulin (IVIG) therapy is effective in preventing CALs^11^, which prompted several clinical researchers to propose several risk factors for IVIG resistance (IVIGR) such as the Kobayashi^12^, Egami^13^ and Sano^14^ scores. However, these scores were not used for predicting IVIGR in countries other than Japan^15^. In addition, they were not useful in predicting CAL formations in terms of sensitivity and specificity^16^. Several methods have been proposed to predict CALs using special substances like N-terminal pro-brain natriuretic peptide (NT-proBNP)^17^, tenaisine^18^ and a cytokine of hepatic growth factor (HGF)^19^ as markers. Conversely, some clinical studies with large samples of KD patients that enabled us predict the presence of CALs has been reported^20,21^.

Recently, the combined therapy with IVIG and steroid hormones is used for KD patients at high risk of IVIGR in daily paediatric clinical practice, based on previously described risk factors^12–14^. For the application of steroid hormones, the following two methods can be used: the oral administration of prednisolone as proposed in the RAISE study^22,23^ and the intravenous administration of methylprednisolone pulse (IVMP)^24^. The reductive effects of these therapies on the occurrence of CALs have shown no significant difference between them^25^. However, some KD patients with CALs are still at high risk of IVIGR even after receiving those therapies^22–24^. Furthermore, in this study, a few patients with CALs at low risk of IVIGR were treated with a standard therapy for KD with and without IVMP. This enabled us to develop a new method of predicting CALs early during admission to help us choose the appropriate therapy for KD patients.

We focused on the pathogenesis of KD, which is associated with some gene^8, 9^ and protein^10^ abnormalities. To analyse the unknown factors, we used a structure equation model (SEM) analysis since we could assume the role of unknown factors in the study using a latent variable. We used a sample score (SS), a factor score for latent variables calculated using the AMOS software, because it is thought to reflect the influence of a latent variable on each subject. We also used a Nnet analysis to predict CAL formation because it is useful for efficiently predicting several events^26^.

Finally, we found the importance of the SS to remarkably discriminate the occurrence of CALs. Thereafter, we introduced an efficient and simple statistical model using the Nnet analysis, which enabled us to predict CALs with sensitivity, specificity and c-index values of 73%, 99% and 0.86, respectively. This method is useful in deciding the second- or third-line therapies using the anti-tumour necrosis factor^27^, cyclosporine^28^, or plasma exchange^29^ for Japanese KD patients on admission after first- or second-line therapy using IVIG with steroid hormones.

## Results

**Figure 1:**
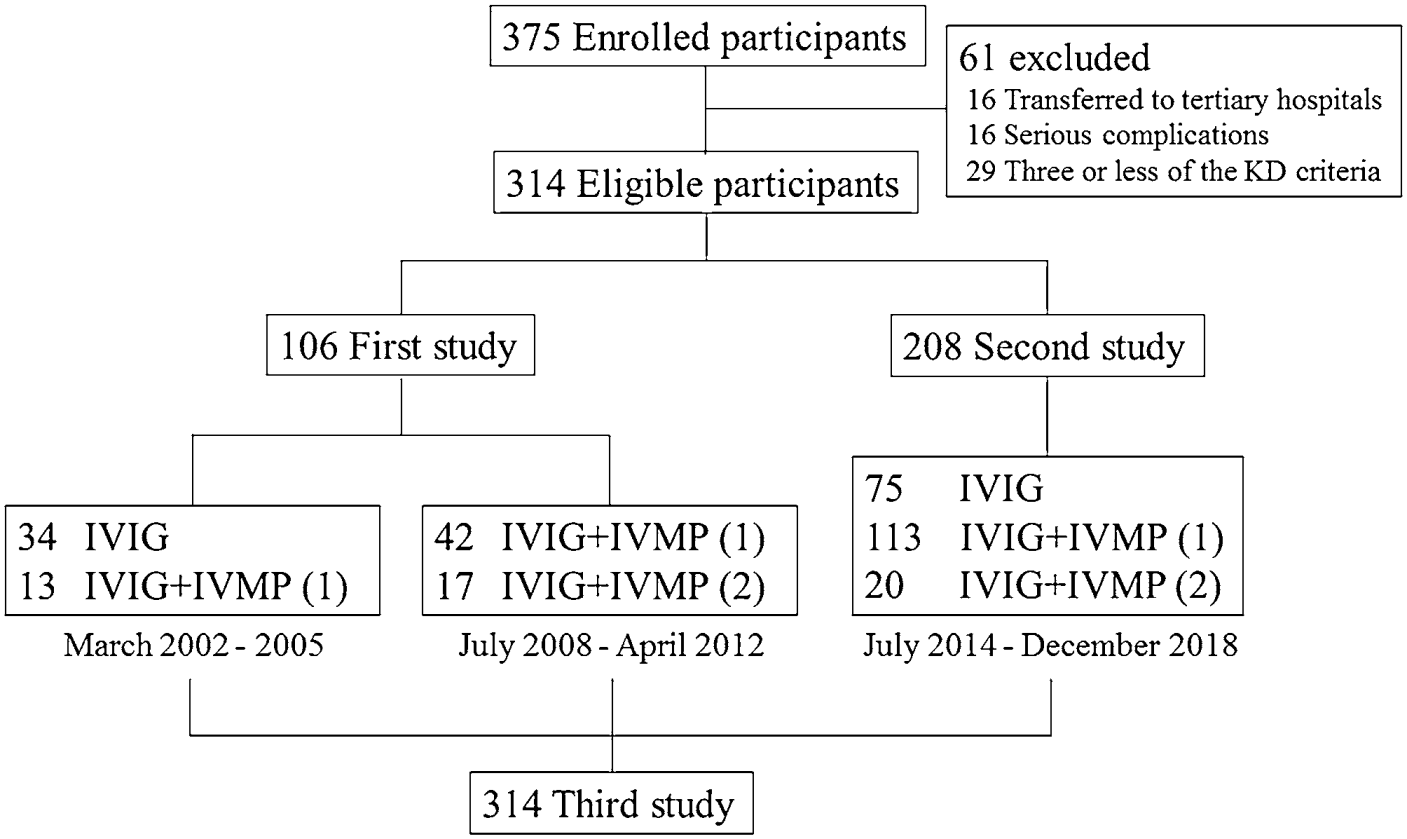
Patient disposition. The numbers in parentheses indicate the number of IVMP treatment courses. *KD* Kawasaki disease, *IVIG* intravenous immunoglobulin, *IVMP* intravenous methylprednisolone pulse.

Amongst the 375 children clinically diagnosed with KD, 61 were excluded. Finally, 314 patients including 106 of 118 in the first study and 208 of 257 in the second one were included (Fig. 1). The patient profiles in the first and second studies are described in Table 1. The value of log urinary β2-microglobulin (u-β2MG/Cr) and the IVIGR value were significantly higher in the first study, whereas the age and coronary artery diameter before the treatment were significantly higher in the second study.

**Table 1:** Comparison of basic characteristics between the first and second studies.

Variables	First study (n = 106)	Second study (n = 208)	*p* values
			Univariate
Age, mean (SD), months	30.5 (2.5)	37.8 (1.8)	0.018*
Sex, male, n (%)	64 (60.4)	121 (58.2)	0.71
Relapse, positive, n (%)	13 (12.3)	31 (14.9)	0.51
IVIGR, positive, n (%)	25 (23.6)	26 (12.5)	0.012*
IVMP times, mean (SD)	0.6 (0.06)	0.74 (0.04)	0.059
Coronary artery diameter, mean (SD), mm	0.62 (0.11)	1.08 (0.07)	0.0004**
WBC, mean (SD), /μL	12,646 (476)	13,289 (340)	0.27
CRP, mean (SD), mg/dL	7.71 (0.47)	7.2 (0.33)	0.38
TB, mean (SD), mg/dL	0.71 (0.06)	0.57 (0.042)	0.084
AST, mean (SD), U/L	58.4 (16.1)	81.9 (11.5)	0.23
Log u-β2MG, mean (SD), µg/gCr	3.56 (0.08)	3.18 (0.06)	0.0002**

Statistical analysis was performed using Student’s *t* test and chi-square test. *p < 0.05, **p < 0.01.

*SD* standard deviation, *n* number, *IVIGR* intravenous immunoglobulin resistance, *IVMP times* number of intravenous methylprednisolone pulse treatment courses, *WBC* white blood cell, *CRP* C-reactive protein, *T-Bill* total bilirubin, *AST* aspartate aminotransferase, *u-β2MG* urinary β2-microglobulin.

## Clinical risk factors associated with CAL formation

### Univariate, linear mixed model and multivariate logistic regression (LR) analyses

In the first study, no variables were associated with CAL formation. However, the linear mixed model revealed that the mean u-β2MG value (*p* = 0.034) and deviation value of C-reactive protein (CRP; *p* = 0.002) were positively associated with CAL formation. Furthermore, the mean aspartate aminotransferase (AST) level positively associated with CALs (*p* = 0.064). The second study showed that the maximum pre-treatment u-β2MG value (*p* = 0.0175) and the occurrence of relapse (*p* = 0.0175) were significantly associated with CALs and the maximum pre-treatment CRP value was positively associated with CALs (*p* = 0.0855). The maximum pre-treatment u-β2MG and CRP values were selected as potential risk factors associated with CAL formation on the basis of the common characteristics of the first and second studies. The LR analysis demonstrated that maximum u-β2MG and maximum coronary diameter before treatment were positively and significantly associated factors (*p* = 0.063 and *p* < 0.0001, respectively).

### Mean structure equation model for path models

Path models were built using age and sex as clinical backgrounds, IVIGR and number of IVIG treatment courses as factors associated with KD^24^, and the maximum pre-treatment u-β2MG and CRP values as elucidated factors.

**Figure 2:**
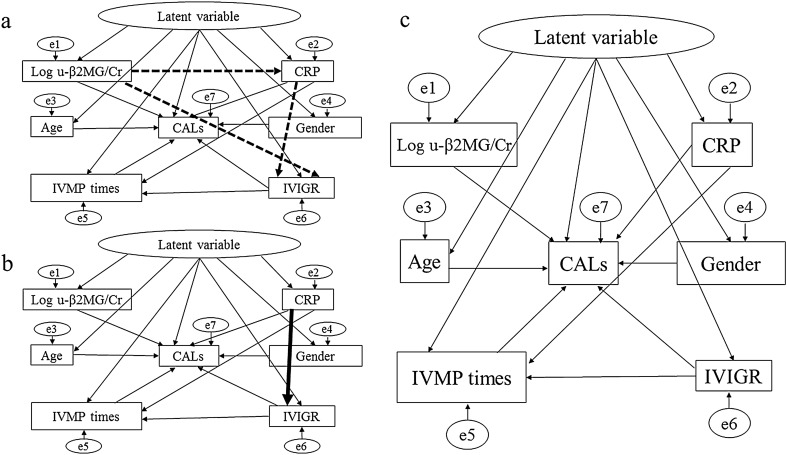
Final path models for each study. (**a**) First study: RMSEA < 0.0001, CFI = 1.000, *R*^2^ for CALs = 1.000, AIC score = 65 and standardised path coefficient of the latent variable for CALs = 0.80. (**b**) Second study: RMSEA < 0.0001, CFI = 1.000, *R*^2^ for CALs = 1.000, AIC score = 61 and standardised path coefficient of the latent variable for CALs = 0.76. (**c**) Third study: RMSEA = 0.032, CFI = 0.98, *R*^2^ for CALs = 1.000, AIC = 65 and standardised path coefficient of the latent variable for CALs = 0.74. The dotted and bold paths denote the differences between (**a**,**c**) and between (**b**,**c**), respectively. *CAL* coronary artery lesion, *CRP* C-reactive protein, *u-β2MG* urinary β2-microglobulin, *IVIGR* intravenous immunoglobulin resistance, *IVMP times* number of intravenous methylprednisolone pulse treatment courses, *e* error.

The path model in the first and second studies showed an excellent fit based on the following values: root mean square error of approximation (RMSEA) of < 0.0001 and < 0.0001; comparative fit index (CFI) of 1.000 and 1.000; *R*^2^ for CALs as 1.000 and 1.000; Akaike information criterion (AIC) score of 65 and 61; standardised path coefficient of the latent variable in terms of total effects on CALs as 0.80 and 0.76 in the first and second studies, respectively (Fig. 2a,b).

### Discrimination of CALs using the SS

**Figure 3:**
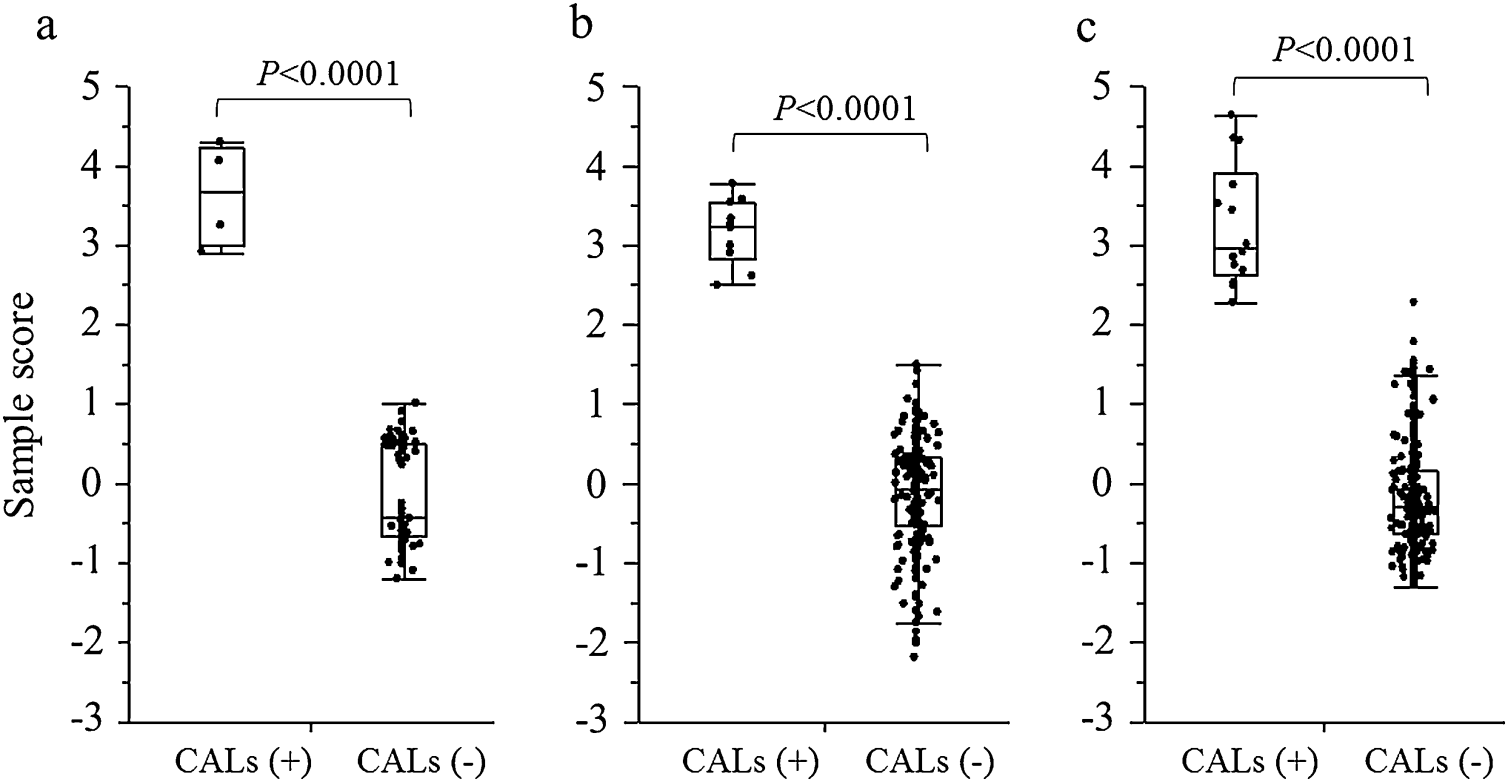
Comparison of sample scores between the patients with CALs and those without CALs in each study. (**a**) First study. (**b**) Second study. (**c**) Third study. The statistical analysis was performed using the Student’s *t* test. CAL, coronary artery lesion.

In the first and second studies, the SSs for the latent variable were significantly higher in patients with CALs than in those without them (both *p* < 0.0001; Fig. 3a,b). The receiver-operating characteristic (ROC) analyses revealed that the cutoff SSs to discriminate the development of CALs were 2.0 and 2.1 in the first and second studies, respectively.

**Figure 4:**
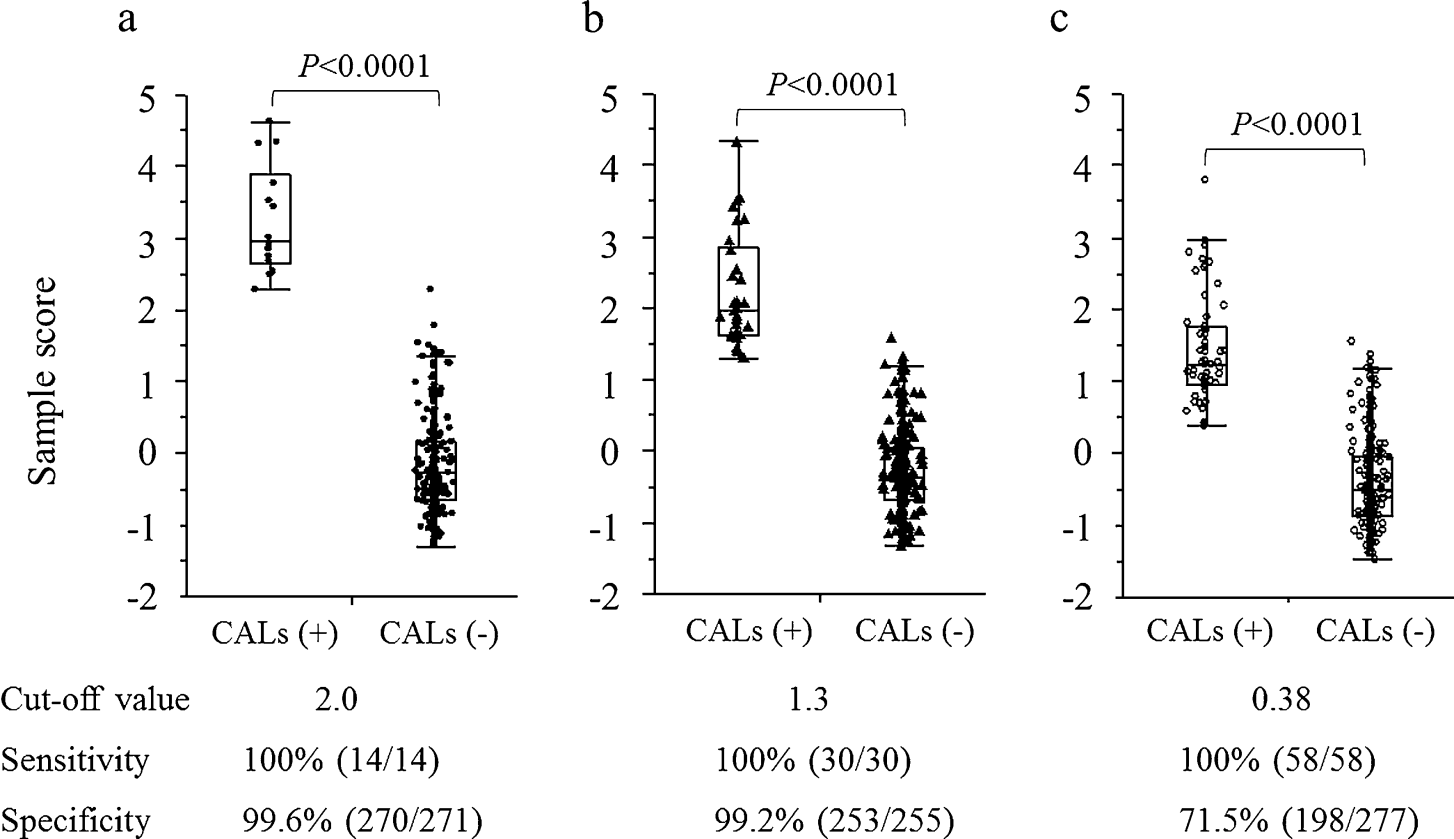
Comparison of sample scores between the patients with CALs and those without CALs when CAL was defined as 3.0 SD (**a**), 2.5 SD (**b**) and 2.0 SD (**c**). The sample scores for the latent variable discriminated CALs best when CAL was defined as 3.0 SD. The numbers in parentheses indicate the numbers of patients. Student’s *t* test was used for the statistical analysis. *CAL* coronary artery lesion.

To confirm whether the potential value of SS for discriminating CALs was true, we integrated all the data in the third study. In addition, an SEM analysis revealed a good fit based on the following values: RMSEA of 0.032; CFI of 0.98; *R*^2^ for CALs as 1.000; AIC score of 65 and standardised path coefficient of the latent variable for CALs as 0.74 (Fig. 2c). In addition, the discrimination for the development of CALs using the SS was remarkable (*p* < 0.0001) and ROC analysis revealed that the cutoff SS was 2.0 (Fig. 3c). Furthermore, we calculated the SS and cutoff values based on the other criteria for CALs, i.e. 2.5 and 2.0 SD, respectively. ROC analysis revealed that the cutoff SS for 2.5 and 2.0 SD were 1.3 and 0.38, respectively. The potential ability of SS to discriminate CALs based on different definitions reduced in a dose-dependent manner according to the criteria of CALs from 3.0 to 2.0 SD (Fig. 4).

To investigate the association of the latent factor with the maximum coronary artery diameters upon admission, another SEM analysis including the maximum values of the coronary artery diameters upon admission was performed. The analysis revealed no direct association between the latent variable and maximal diameters of the coronary arteries upon admission (standardised path coefficient, 0.095; *p* = 0.25).

### Neural network analysis

**Figure 5:**
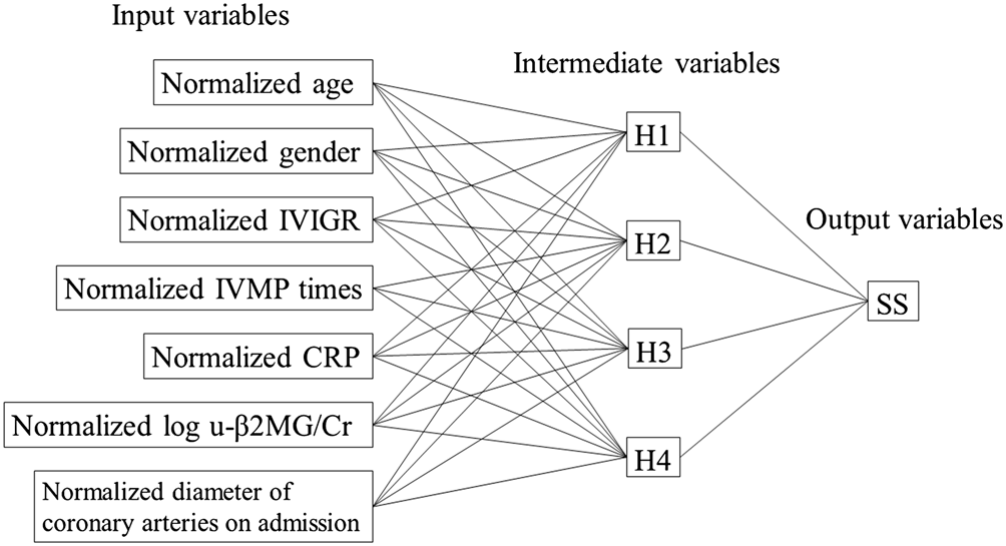
Artificial neural networks for predicting CALs using sample scores. Intermediate variable (H1) was denoted as the formula shown in SI Table S1. *IVIGR* intravenous immunoglobulin resistance, *IVMP times* number of intravenous methylprednisolone pulse treatment courses, *CRP* C-reactive protein, *u-β2MG* urinary β2-microglobulin, *SS* sample score.

The Nnet analysis included seven input variables comprising the maximum diameters of the coronary arteries on admission and six other variables used in the SEM analysis. All these variables were normalized as previously described^30^. The output variable was the SS for the development of CALs (Fig. 5). Subsequently, we obtained a statistical model with four nodes in the intermediate layer, an overfit penalty of 0.02, *R*^2^ of 0.89 and *R*^2^ of 0.64 by the five-fold cross-validation method, although there were a few outlier patient (SI Fig. S1). The sensitivity and specificity for the prediction of CAL development were 73% and 99%, respectively, when the cutoff SS value was 2.0.

The combined data of LR and Nnet analyses did not increase the sensitivity and specificity of 73% and 99%, respectively (Table 2). In a new data set consisting of 38 KD patients from 2019 to 2020 at our hospital, the sensitivity and specificity were 50% and 100%, respectively.

**Table 2:** Comparison of sensitivity, specificity and c-index among the three statistical models.

	(A) Logistic regression analysis model data	(B) Neural networks analysis model data	Combined A and B model data
Sensitivity	36% (4/11)	73% (8/11)	73% (8/11)
Specificity	99% (232/234)	99% (232/234)	99% (231/234)
C-index	0.68	0.86	0.86

The numbers in parentheses indicate the number of patients.

### Discussion

We developed a new method of predicting CALs in KD using SEM and Nnet analyses. This is the first study to show the causal associations between a latent factor of CALs and the presence of CALs.

Several articles have dealt with the prediction of CALs; some of which presented laboratory data on special substances like NT-proBNP^17^ or a cytokine of HGF^19^, whereas others focused on clinical data^20,21^. The method used in this study is superior to all the previous methods described in former studies. This is because it is the first to indicate the usefulness of SS, a latent variable, predicting CALs in KD patients. Although the predicting ability in this case was slightly inferior to that of the previous report in terms of c-index^21^, we believe that there are some advantages compared to the previous reports. First, the predicting ability of LR analyses using measurable variables was fair in previous studies^20,21^, whereas it was poor in this case. We could attribute this to racial differences. Similar discrepancies were often highlighted in the risk score for IVIGR^15^ in KD patients. In our study, the SS used in Nnet analysis was a latent factor obtained by AMOS. Moreover, the latent factor was supposed to be a reflection of the predisposition to KD patients; thus, some genes associated with the predisposition might be more important than the real measured data in the Japanese population for the prediction of CALs in KD. Further studies are necessary to elucidate the details.

Second, measured only three parameters in daily clinical practice, i.e. serum CRP level, u-β2MG/Cr ratio, and the maximal diameters of the coronary arteries upon admission. The other necessary information for our method was easily obtained upon admission like age, sex, IVIGR (Sano score) and number of IVMP treatment courses due to the risk based on IVIGR scores.

Third, we agree that a sensitivity of 73% and specificity of 99% with a c-index of 0.86 is not exceptional; however, this is thought to be better than the IVIGR scores, because we can directly predict the formation of CALs instead of formulating the clinical process of IVIGR patients. Since we found some outlier patients in the analysis, it is speculated that the sensitivity can be increased if we find more appropriate factors for the input layers of Nnet analysis. Further studies are needed in the future.

This study included data from two retrospective studies. In the first study, no significant differences between the investigated parameters were observed. This was supposed to be derived from the different measurers of coronary artery diameters among the patients. However, the use of a mixed linear model eliminated this problem and revealed that the maximal serum CRP level and u-β2MG/Cr ratio were statistically significant factors because the analysis reduced the degree of repeated measurement errors. In the second study, the same measurer obtained the coronary artery diameters that enabled us to identify the important parameters using the Student’s *t* test and chi-square test. These results confirmed the importance of standardisation of coronary artery measurements. Several reports have indicated that maximal CRP values are associated with CAL formation^20,21^. In contrast, no report has identified the importance of u-β2MG/Cr values. However, these findings might be supported by the observation that suggests the importance of u-β2MG/Cr values for IVIGR^31^ because IVIGR is a risk factor of CAL formation^11^. β2MG is involved in the cytokine networks^32^; therefore, the relationship between u-β2MG/Cr values and the development of KD needs to be investigated, particularly from the point of view of abnormal innate immunity, which is associated with KD^7^.

Recently, several genes have been associated with KD^8,9^, suggesting the presence of unknown factors associated with its pathogenesis. Using standard statistical methods, the effects of unknown factors could be investigated. Thus, we used the SEM analysis to clarify the role of a latent factor in CAL formation.

The SEM was devised in the 1970s^33^ and was routinely used for analysing social and psychological aspects of problems^33^ because it involves several complicated and unknown factors. However, we sometimes encounter the complicated relationships between the clinical parameters even in the field of paediatric clinical medicine, suggesting that the SEM model may be suitable in the analysis of statistical data obtained from our daily clinical practice.

In fact, using SEM analysis, we clarified the role of serum IgG3 levels in the clinical severity of pandemic influenza H1N1pdm, an unknown latent factor^34^. Therefore, we used the SS as a latent variable in this study because it reflects the influence of the pathogenesis of KD on each patient when we set a path from the latent variable to CAL formation. Since the efficiency of CAL discrimination by SS was associated with the cutoff SS by the ROC analysis, we thought that the SS can be used for predicting CAL formations in KD patients. Indeed, our hypothesis was proven true by the fact that CAL formation was remarkably discriminated by the SS (*p* < 0.0001). In addition, the presence of the causal association between the SS and CAL formation was confirmed by our finding that the potential value of SS to discriminate CALs based on the different definitions reduced in a dose-dependent manner according to the criteria for CAL from 3.0 to 2.0 SD.

Based on our data, we speculate that unknown factors possibly relevant to predisposition, representative of latent variables, are important for CAL formation in Japanese KD patients. To prove this hypothesis, we eliminated a latent factor that led to a severe reduction in the *R*^2^ value for CALs from 1.000 to 0.020 (data not shown). This also suggests the importance of the SEM analysis in creating a new statistical model for predicting CAL formation at least in Japan.

In addition, no evidence of association was found in the additional SEM analysis between a latent variable and the maximal diameters of the coronary arteries upon admission, suggesting the possibility of another mechanism being involved in the previously reported association of the maximal coronary artery diameters upon admission with CAL formation^16^. Further studies are required on this issue.

Recently, the Nnet analysis was reported to be useful in predicting several events^35^. In fact, we previously reported on this aspect of Nnet, using it to predict future bone mineral density and annual bone loss rate in postmenopausal women^30^. This experience led us to use Nnet analysis to predict the occurrence of CALs in the third study. The analysis enabled us develop a good and simple statistical model for *R*^2^ of 0.89 and *R*^2^ of 0.64 in the five-fold cross-validation study to predict CALs with a sensitivity and specificity of 73% and 99%, respectively, using a cutoff SS of 2.0. The five-fold cross-validation study is proposed to reduce the possibility of overfitting, which is recommended in a clinical study using relatively small number of patients. However, this method is not perfect.

To confirm whether our methods have translational impacts, we investigated a new data set involving 38 KD patients from 2019 to 2020 at our hospital, including two patients transferred to the tertiary referral hospital because we had no KD patients with CALs from 2019 to 2020. Using the combined method of Nnet and LR analyses, it revealed sensitivity and specificity of 50% and 100%, respectively. These data did not contradict the retrospective model data in this study, despite the few patients. On the basis of these data, we believe that our protocols for predicting CALs in KD patients using the Nnet analysis have translational impacts on the field of clinical paediatrics.

For the Nnet analysis, we added the maximal coronary artery diameters upon admission to the input variables. This is supported by a previous report^16^ that suggested the important role of the maximal coronary artery diameters upon admission for the accurate prediction of CAL formation.

The influence of this study’s IVMP design on the evaluation of sensitivity and specificity may be questioned. To answer this, we investigated the effect of elimination of IVMP in the AMOS study, which increased the value of SS to 2.3. Subsequently, we performed Nnet analysis again to select the new program and found cross-validation values of *R*^*2*^ (0.84 and 0.66). It demonstrated a sensitivity and specificity of 55% and 99%, respectively and the former value was < 73% in this study. These data suggested the influence of IVMP therapy on the prediction of CALs. We speculated the relationship between these findings and the evidence of the usefulness of IVMP to prevent CALs.

The timing of the factors to sample and input is another aspect to consider. For u-β2MG/Cr and CRP values, we used the maximal values before starting the first-line therapy in our hospital. For the diameter of coronary arteries, we used the values before starting the first-line therapy and also the maximal values during admission in our hospital. For the new data set, we added the two patients in the second study with CALs based on the reports from the tertiary referral hospital. For the needed number of IVMP treatments, we are able to decide based on IVIGR scores on admission. Therefore, we can decide all the input variables for this method before the first-line therapy. Thus, our method can help the doctors predict the occurrence of CALs upon admission and decide whether they should opt for the second- or third-line therapy for KD earlier. Also, we conclude that the findings in this study are necessary in the clinical setting as the results predicted CALs to have a sensitivity, specificity and c-index of 73%, 99% and 0.86, respectively.

There are several limitations of using the Nnet method. One is the problem of overfitting. To avoid this, we used a five-fold cross-validation method to select a program when *R*^2^ values on cross-validation were > 0.64 (r = 0.8). Moreover, we investigated a new data set to reconfirm the translational impacts of our method on the field of paediatric clinics. However, these data need to be further investigated because the new data set had fewer patients. Another limitation is the possible presence of other suitable variables for the input layer of Nnet because of the presence of few outlier patients to predict SS for CALs.

There are also some limitations to using the AMOS method. One is the SS that was used to predict CALs. Since the SS was calculated from the data of each sample with the mean value and deviations from the mean by AMOS, SS might differ with hospitals, depending on the clinical severity of KD patients and the clinical role of each hospital, such as that of tertiary hospitals.

Another limitation could be the treatment method, because its process is different for patients from March 2002 to 2005 and patients from July 2008 to April 2012 studies, even though we tried to correct the bias statistically through AMOS and Nnet. This limitation is thought to be derived using a retrospective study design and could be clarified by a prospective study in the future.

Finally, a future confirmatory study with a large number of Japanese patients using these methods is necessary since the incidence of CALs in this study was approximately 5%, which resulted in only 14 KD patients and CALs for analyses.

In conclusion, we developed a good and simple statistical model with remarkable accuracy in discriminating CALs using common parameters that are used in the field of clinical medicine. This method is useful for deciding the time and type of therapy for Japanese KD patients to prevent CAL formation in the future.

## Methods

We conducted a retrospective investigation using the opt-out method of our hospital. The ethics review board of the Minoh City Hospital approved the study and also waived the need for informed consent. All methods were performed per the Declaration of Helsinki and relevant guidelines.

## Subjects

We included 375 children (202 boys, 152 girls and 21 others (gender not known)) who were clinically diagnosed with KD and admitted to the Minoh City Hospital between March 2002 and 2005, between July 2008 and April 2012 (the first study) and between July 2014 and December 2018 (the second study). Patients with < 4 of the diagnostic criteria for KD (n = 29), patients who were transferred to a tertiary referral hospital (n = 16), or had serious complications (n = 16) like anaphylaxis and drug-induced hypersensitivity syndrome were excluded. Finally, 314 children (185 boys and 129 girls) were deemed eligible for this study. Patients with at least two of the following three variables were classified into the high-risk group who might be non-responsive to IVIG therapy: CRP level ≥ 7 mg/dL, total bilirubin (T-Bill) level ≥ 0.9 mg/dL and aspartate aminotransferase (AST) level ≥ 200 U/L (Sano score)^14^. The remaining patients were classified into the low-risk group. In the first study, we used IVIG (2 g/kg) with IVMP (30 mg/kg) therapy for patients in the high-risk group (n = 13) and only IVIG therapy for those in the low-risk group (n = 34) between March 2002 and 2005. On the other hand, we used IVIG with two doses of IVMP therapy for patients in the high-risk group (n = 17) and IVIG with IVMP for those in the low-risk group (n = 42) between July 2008 and April 2012. In the second study, some patients with CRP levels of ≥ 7 mg/dL were treated with IVIG with IVMP therapy (n = 113) and those with CRP levels of < 7 mg/dL were treated with only IVIG therapy in the low-risk group (n = 75), whereas those in the high-risk group (n = 20) were treated with IVIG with two doses of IVMP therapy (Fig. 1).

### Echocardiography and laboratory tests

To evaluate coronary artery dilation, two-dimensional echocardiographic measurements of coronary artery diameters were performed. In detail, the diameters of the left main coronary trunk artery (LMT), proximal left anterior descending coronary artery (LAD) and proximal right coronary artery (RCA) were measured at three time points: before the treatment, immediately after the treatment and at hospital discharge. The diameters of the coronary arteries were expressed as *z* scores calculated using the least mean square method^36^. We defined CALs as a maximum *z* score of ≥ 3.0 for the LMT, LAD or RCA. Laboratory tests were performed at the above mentioned three time points. Serum constituents were measured with the standard techniques used at Minoh City Hospital. The levels of u-β2MG were measured using a latex immunoassay kit (Wako, Osaka, Japan).

### Study design

We performed a retrospective evaluation of KD patients to elucidate the risk factors of CAL formation. The investigated variables were age; sex; white blood cell count; levels of CRP, T-Bill, AST and u-β2MG/Cr; presence of relapse; IVIGR (Sano score) and number of IVMP treatment courses. In the first study, the diameters of the coronary arteries were measured by several physicians, which may have caused a measurement variation in addition to several missing clinical data. Therefore, we used a linear mixed model in the first study. In contrast, we used the Student’s *t* test and chi-square test in the second study because the coronary artery diameters were measured by only one physician with few missing clinical data. We selected variables to identify the risk factors of CAL formation from the first and second studies in terms of the statistical significance and tendency of the common characteristics of CALs. On the other hand, we investigated the possibility of usefulness of neutrophil counts, sodium and albumin by logistic regression analyses in our preliminary studies but we did not find significant effects on predicting CALs. In this investigation, we did not include hematocrit because we were not able to find it as the predictor of the occurrence of CALs by the tool of pubmed central. The SEM was used to build path models with a latent variable. In the first study, paths between the variables were selected when they showed a significant correlation of *p* < 0.002 with correlation coefficients of > 0.3. In the second study, we selected the path that showed the coefficient values of the maximal standardised path between a latent variable and CALs on the basis of a significant correlation of *p* < 0.0001, with correlation coefficients of > 0.3. Finally, we integrated all the data of the first and second studies to investigate the possibility of predicting the development of CALs using the SEM (the third study). In the third study, the criteria for significant correlation of *p* < 0.0001 with correlation coefficients > 0.33 were used. We supposed that the correlation coefficient no less than 0.3 is clinically more important than the *p* values. This explains why the cutoff *p* values were different among the first and second studies. In all three studies, we examined the statistical significance between the development of CALs and the SSs for the latent variable using Student’s *t* tests. Thereafter, a ROC analysis was conducted to determine the cutoff SS for discriminating the CALs.

In the Nnet, we used the data on the diameters of the coronary arteries upon admission in addition to the same variables selected as those in the SEM in the third study. Two to four nodes in the intermediate layer from 0.01, 0.02 and 0.04 overfit penalties were selected. For the selected statistical model, the criteria of the best measured *R*^2^ ≥ 0.81 (r = 0.9) and *R*^2^ ≥ 0.64 (r = 0.8) for five-fold cross-validation method were used.

Furthermore, LR analysis was performed using age and sex as clinical backgrounds, IVIGR and number of IVIG treatment courses as factors associated with KD^24^ and the maximum u-β2MG, CRP values and maximal coronary diameter before the treatment as elucidated factors in the third-study patients. Furthermore, we investigated the usefulness of combined data of LR (real measured data) and NNet analyses (predicted scores by AMOS). We considered it positive when one of the analyses showed the positive occurrence of CALs.

Lastly, we investigated a new data set to confirm whether our methods have translational impacts. This set included 38 KD patients from 2019 to 2020 at our hospital. Because there were no patients with CALs in the new samples, we included two patients who were transferred to the tertiary hospital and had CALs but were not analysed in the previous study based on the exclusion criteria. One patient was treated with IVIG with two times of IVMP therapy in Minoh City Hospital; however, fever persisted after the therapy. Then, we transferred the patient to the tertiary referral hospital and he was treated with plasmapheresis. The other one was treated with IVIG with IVMP therapy in Minoh City Hospital; however, five KD symptoms persisted in spite of the therapy. Thus, we transferred the patient to a tertiary referral hospital and she was treated with additional IVIG with oral cyclosporin.

### Statistical analyses

*p* < 0.05 and 0.05 < *p* < 0.1 were accepted as statistically significant and prone, respectively. The statistical significance of the path models was judged from the following criteria: RMSEA < 0.05, AIC score < 70, CFI score > 0.95 and *R*^2^ score > 0.95. The JMP version 8.0 software (SAS Institute, Cary, NC) was used for the Student’s *t* test, chi-square test and Nnet analysis. AMOS 23.0 (IBM-SPSS, USA) and SPSS version 23.0 (IBM Corp, Armonk, NY, USA) were used for the SEM and linear mixed model, respectively.

## Data availability

The datasets analysed during the current study are available from the corresponding author on reasonable request.

## Supplementary information

Supplementary file1 (PDF 186 kb)
