# Supplementary material for: Structure equation model and neural network analyses to predict coronary artery lesions in Kawasaki disease: a single-centre retrospective study
Source: Sci Rep. 2020 Jul 17;10:11868. doi: 10.1038/s41598-020-68657-0 (PMC7368009; doi:10.1038/s41598-020-68657-0)
Supplement: Supplementary file 1 — Supplementary file1 (PDF 186 kb) [file 41598_2020_68657_MOESM1_ESM.pdf]

SUPPLEMENTARY INFORMATION

Structure equation model and neural network analyses to predict coronary artery lesions in Kawasaki disease: A single-centre retrospective study

Junji Azuma, Takehisa Yamamoto*, Motoaki Nitta, Yasuhiro Hasegawa, Eri Kijima, Tunesuke Shimotsuji and Yoshimi Mizoguchi

Department of Paediatrics, Minoh City Hospital, 5-7-1 Kayano, Minoh, Osaka 562-0014, Japan

*Corresponding author: t.yamamoto@minoh-hp.jp

Supplementary Figure S1. Correlations between the actual and measured sample scores using the artificial neural networks.

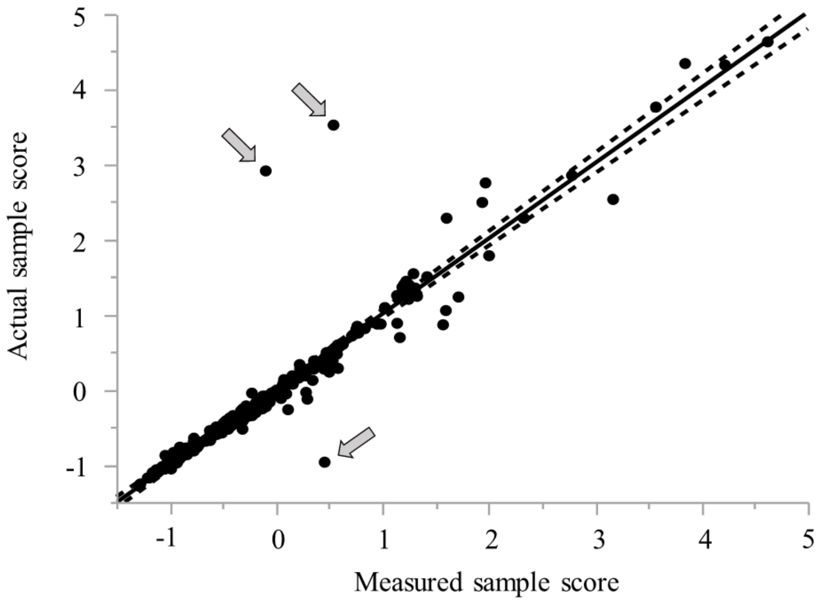

The arrows indicate the outlier patients.

Supplementary Table S1. The formula of intermediate variable (H1).

Squish (3.16967192227212 + 1.40229269682789 * ((normalized age - 0.294597610302664) /0.151933439389203) + 5.44359850050827 * (normalized gender - 0.571337579617835) /0.394215515798604) + 2.14592348919733 * normalized risk in number - 0.229936305732485) / 0.295539787335191) + -12.5796298495775 * (normalized risk in number steroid pulse therapy in number - 0.40828025477707) /0.257280390161389) + -9.18797354477239 * (normalized CRP values - 0.316992990319966) /0.144575821166859) + 6.4299654932182 * (normalized risk log u-b2Mg/Cr- 0.500210847551672) /0.151568074013966) + 3.07357471285414 * (SD vales of maximal coronary artery diameter before therapy) - 0.949888456195652) / 0.978890132842702. Squish denoted a way of calculation as follows, 1/1+exp (-X).
